# Thirty-month results after the treatment of post-LASIK ectasia with allogenic lenticule addition and corneal cross-linking: a case report

**DOI:** 10.1186/s12886-018-0967-z

**Published:** 2018-11-12

**Authors:** Meiyan Li, Dong Yang, Feng Zhao, Tian Han, Meng Li, Xingtao Zhou, Katherine Ni

**Affiliations:** 1 Key Lab of Myopia, Ministry of Health, Department of Ophthalmology, EYE & ENT Hospital of Fudan University, 83 Fenyang Road, Shanghai, 200031 China; 2 School of Medicine, New York University, New York, USA

**Keywords:** Lenticule addition, CXL, Post-LASIK ectasia

## Abstract

**Background:**

To report a first case of lenticule addition and corneal cross-linking for post-LASIK ectasia with associated corneal thinning.

**Case presentation:**

Lenticule addition followed by corneal cross-linking was performed on the left eye of a patient with post-LASIK ectasia. Postoperatively, the corneal power and elevation were stable with a remarkable improvement in corneal thickness, and the lenticule had merged with the adjacent corneal stroma at 30 months follow-up. The patient’s corrected distance visual acuity gained two lines.

**Conclusions:**

This case provides a potential treatment option for patients with keratectasia and keratoconus in association with thin corneal thickness (less than 400 μm) and may provide the benefit of delaying or avoiding the need for keratoplasty, which has its own associated complications and is limited by the availability of donor corneas.

## Background

Post–laser-assisted in situ keratomileusis (LASIK) ectasia is a potential complication of LASIK procedures, and these patients have subsequent progressive corneal thinning and bulging with keratoconus-like topography [1]. The refractive error induced by the ectatic cornea is initially managed with either spectacles or contact lenses. However, when the ectasia progresses to the point where contact lenses are inadequate for vision correction, then surgical intervention may be considered [1, 2].

In the last 15 years corneal cross-linking (CXL) emerged as an effective technique in halting progressive keratoconus, which has been confirmed by many studies [3–5]. Later, further clinical studies showed good results also in patients with secondary corneal ectasia after refractive surgery, and therefore it is currently considered as one of the first surgical options in these cases [6, 7]. With recent developments in laser technology, small incision lenticule extraction (SMILE) has gained widespread acceptance. In addition, application of stromal lenticules extracted by SMILE surgery has been reported to be used for the treatment of hyperopia [8], corneal perforation [9], keratoconus [10], and post-LASIK ectasia [11].

In this study, we present a case to investigate long-term results following lenticule addition and corneal cross-linking for post-LASIK ectasia with associated corneal thinning.

## Case presentation

**Fig. 1:**
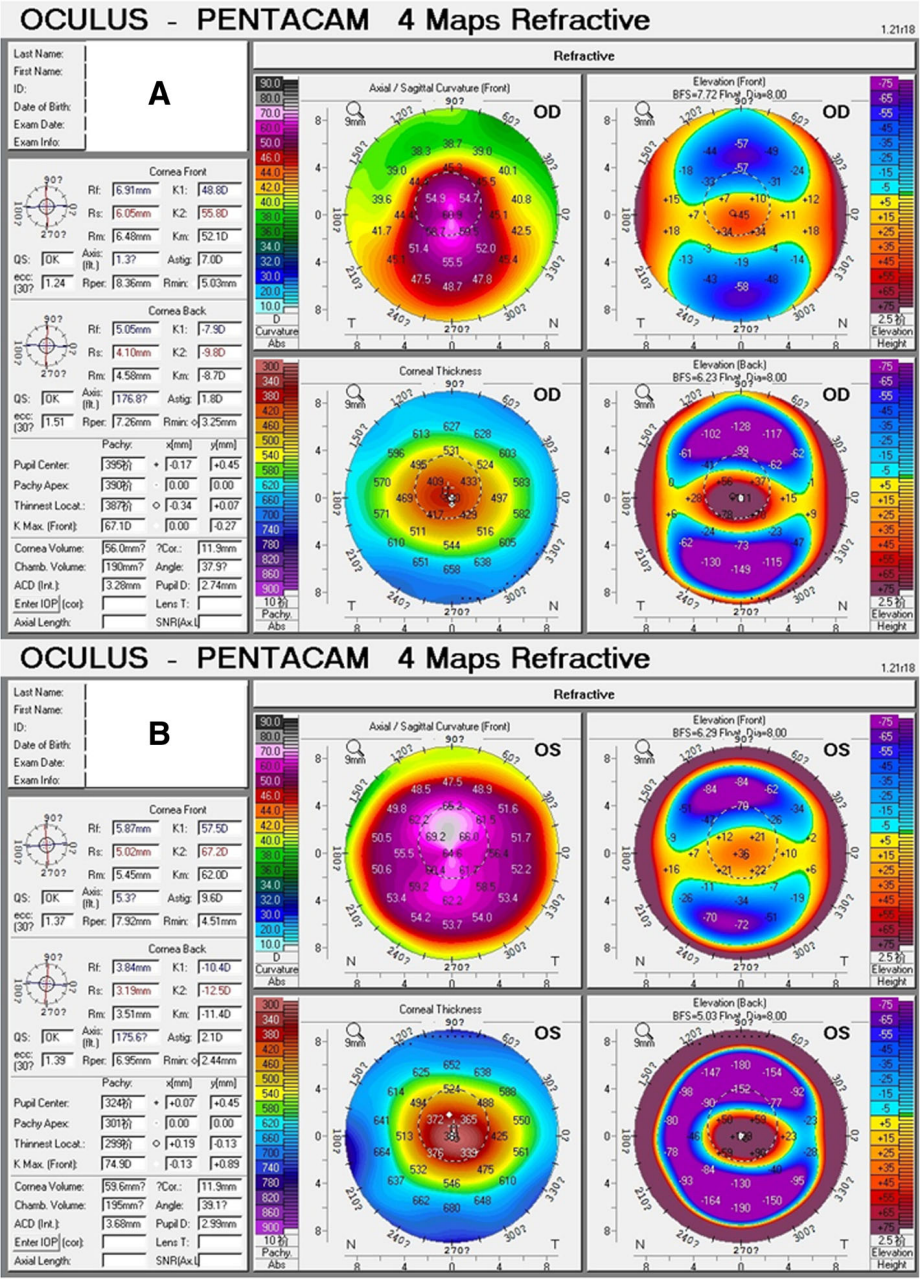
Initial Scheimpflug-based corneal topography examination of the right eye (**a**) and the left eye (**b**) showing sagittal map, pachymetry map, anterior elevation map, and posterior elevation map

A 26-year-old man who underwent bilateral LASIK at 2009 developed bilateral post-LASIK keratectasia 2 years later. In April 2015, he came to our clinic for treatment of the left eye due to in tolerance to rigid gas permeable contact lenses (RGP). On examination, he had an uncorrected distance visual acuity (UDVA) of 20/200 in the right eye, which improved to 20/50 with a refractive correction of − 3.25 / -5.00 @ 160 degrees. His left eye had a UDVA of 20/200, which improved to 20/63 with a refractive correction of − 3.50 / -5.50 @ 100 degrees. Central corneal pachymetry was 395 μm in the right eye and 324 μm in the left eye. Corneal topography showed an inferior steepening (difference inferior-superior of around 13.40 D) in the right eye with a Sim K of 48.80 D at approximately 1.3 degrees and 55.80 D at approximately 91.3 degrees, and a diffuse corneal steepening more noticeable in the superior cornea (difference superior-inferior of around 4.80 D) with a Sim K of 57.50 D at approximately 5.3 degrees and 67.20 D at approximately 95.3 degrees in the left eye (Fig. 1a&b). As the patient’s corneal thickness was exceedingly thin at less than 400 μm, a CXL procedure was not recommended [12]. A lenticule addition procedure was approved by the Ethics Committee of the Fudan University EENT Hospital Review Board. After a written informed consent from the donor and recipient patients, the donor patient received blood testing for human immunodeficiency virus, hepatitis B and C viruses, blood glucose, rapid plasma reagin, and Treponema pallidum particle agglutination, and all results were normal. After that, the recipient patient underwent lenticule addition in his left eye. A maximum (central thickness) of 77 μm and minimum (peripheral thickness) of 10 μm lenticule was obtained the same day from a myopic SMILE of − 0.75 / -2.75 @ 180 degrees using the VisuMax femtosecond laser (Carl Zeiss Meditec, Jena, Germany). A sinskey hook was used to open the edge of the original flap, and a blunt spatula was used to lift the flap. The fresh lenticule was immediately added into the stroma, and carefully centered onto the apex of the cornea. Then the flap was repositioned and a bandage contact lens was applied.

Postoperative topical medication consisted of levofloxacin 4 times daily for 3 days, fluorometholone 0.1% 8 times daily, tapered to once daily over a period of 24 days, and a tear supplement 4 times daily for 1 month.

Four months after lenticule addition, the patient underwent CXL procedure of the left eye, as he had a sufficient corneal thickness of 412 μm. ParaCel (Avedro, Waltham, MA, USA) containing 0.25% riboflavin-5-phosphate, hydroxypropyl methylcellulose, sodium edetate, trometamol, benzalkonium chloride, and NaCl in a corneal epithelial trephine (Model 52503B; 66 Vision-Tech, Suzhou, China) was used to completely cover the cornea for a total of 4 min. The cornea was then rinsed completely with VibeX Xtra (Avedro) containing 0.25% riboflavin-5-phosphate and NaCl, and VibeX Xtra was used in the corneal epithelial trephine for a total of 6 min. After using the epithelial trephine, the cornea was rinsed completely with balanced salt solution (BSS). Ultraviolet treatment was conducted using the KXL System (Avedro). The treatment protocol consisted of pulsed illumination for 1 s using 45 mW/cm^2^ for a surface dose of 7.2 J, and frequency of the pulses was 50 Hz. The ultraviolet treatment procedure lasted for 5 min and 20 s. The cornea was then rinsed completely with BSS, and a bandage contact lens was applied. Antibiotic drops were administered for 1 week, and fluorometholone 0.1% were applied for 16 days (four times a day initially, then reduced once every 4 days). A tear supplement was also prescribed for 4 times per day for 1 month.

**Fig. 2:**
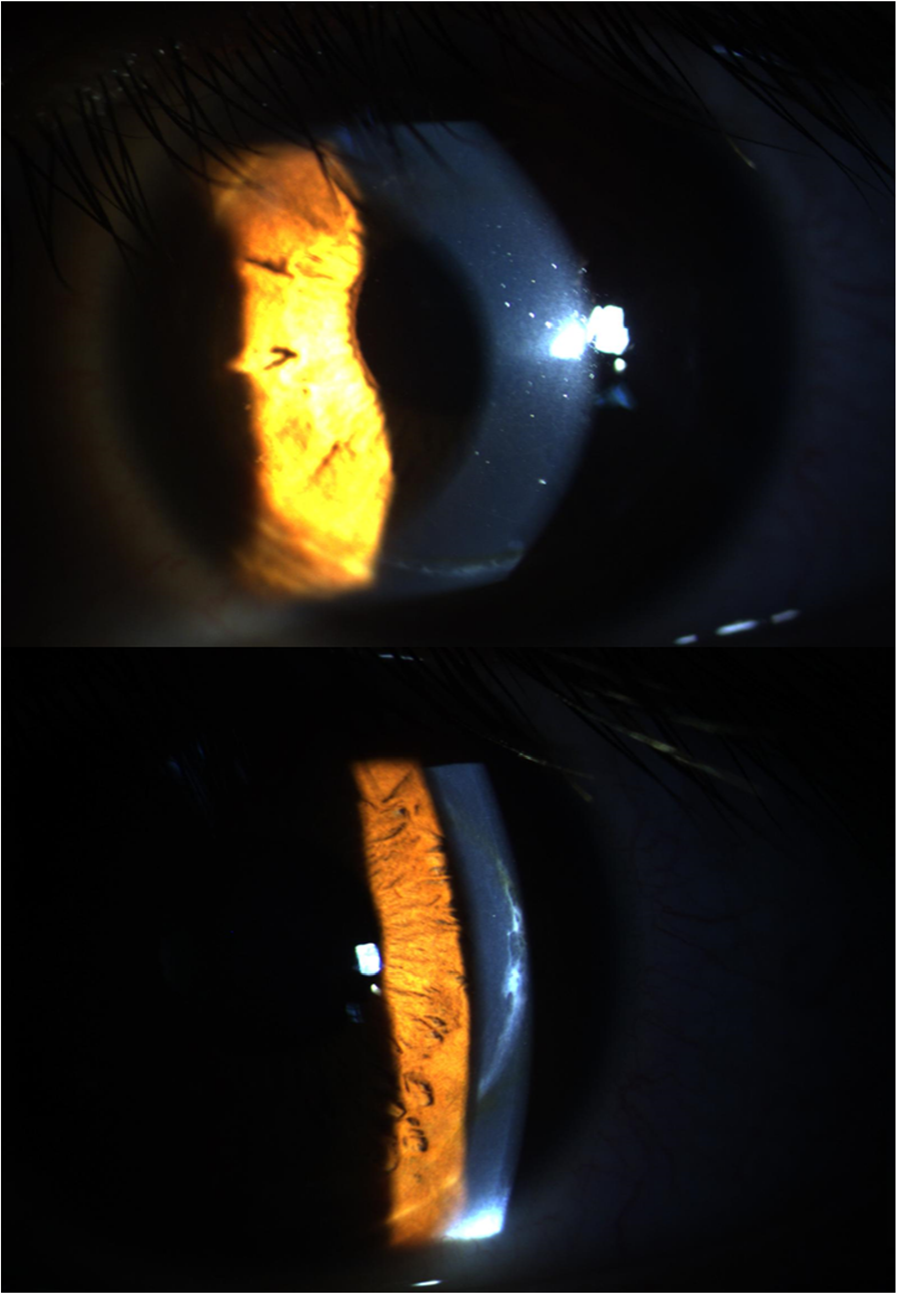
Slitlamp photograph showing opacity at the flap edge at 30 months postoperatively

The patient had a stable UDVA of 20/125 and a best spectacle-corrected distance visual acuity (CDVA) of 20/40 with unchanging refraction of − 5.00 / -6.00 @ 100 degrees from 1 month after lenticule addition to 30 months postoperatively in his left eye. There were no visual abnormalities (such as halos or diplopia), corneal haze, or rejection observed under slit-lamp examination throughout the follow-up period (Fig. 2).

**Fig. 3:**
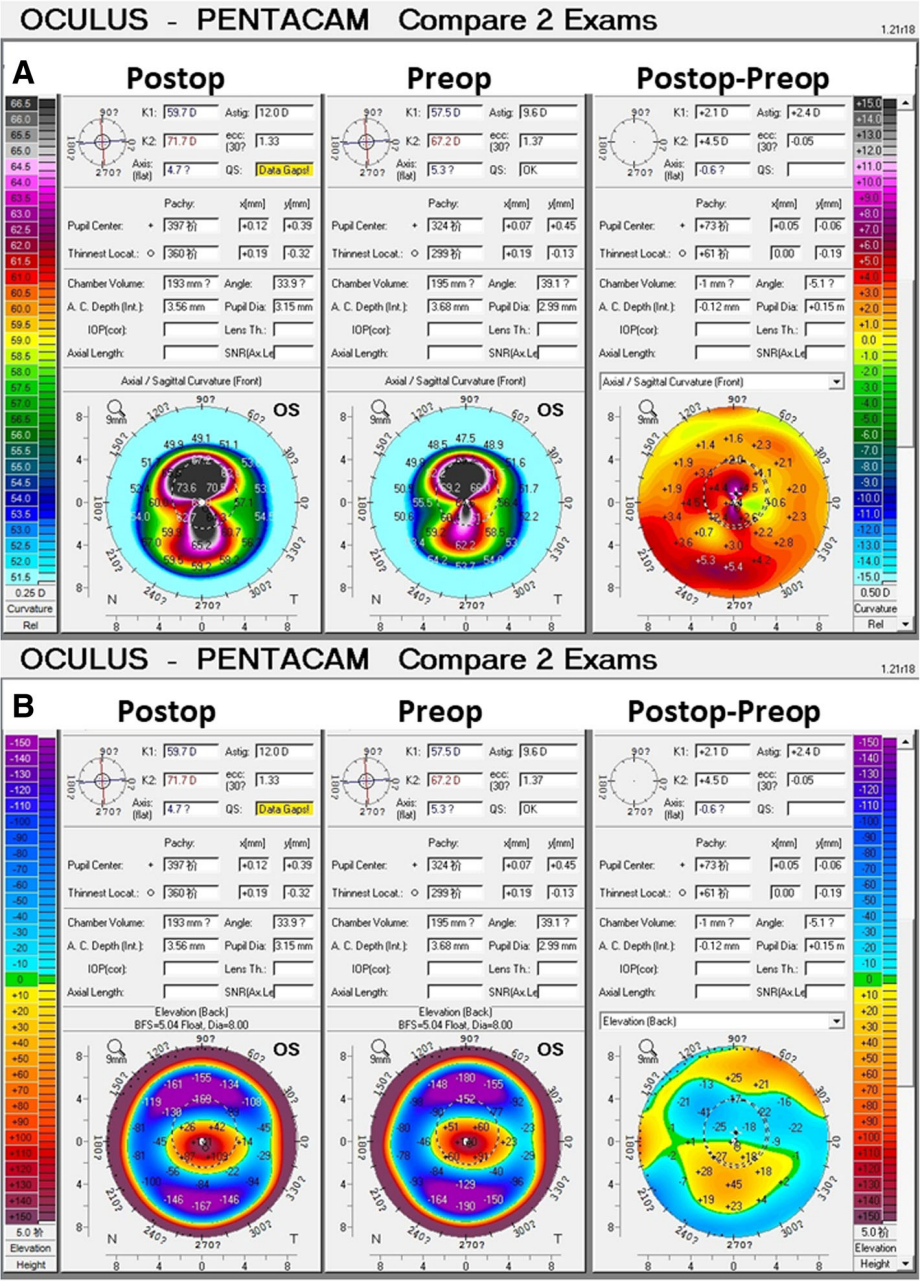
30 months of postoperative sagittal map, preoperative sagittal map, and comparative maps of the left eye (**a**). 30 months of postoperative posterior elevation map, preoperative posterior elevation map, and comparative maps of the left eye (**b**)

**Fig. 4:**
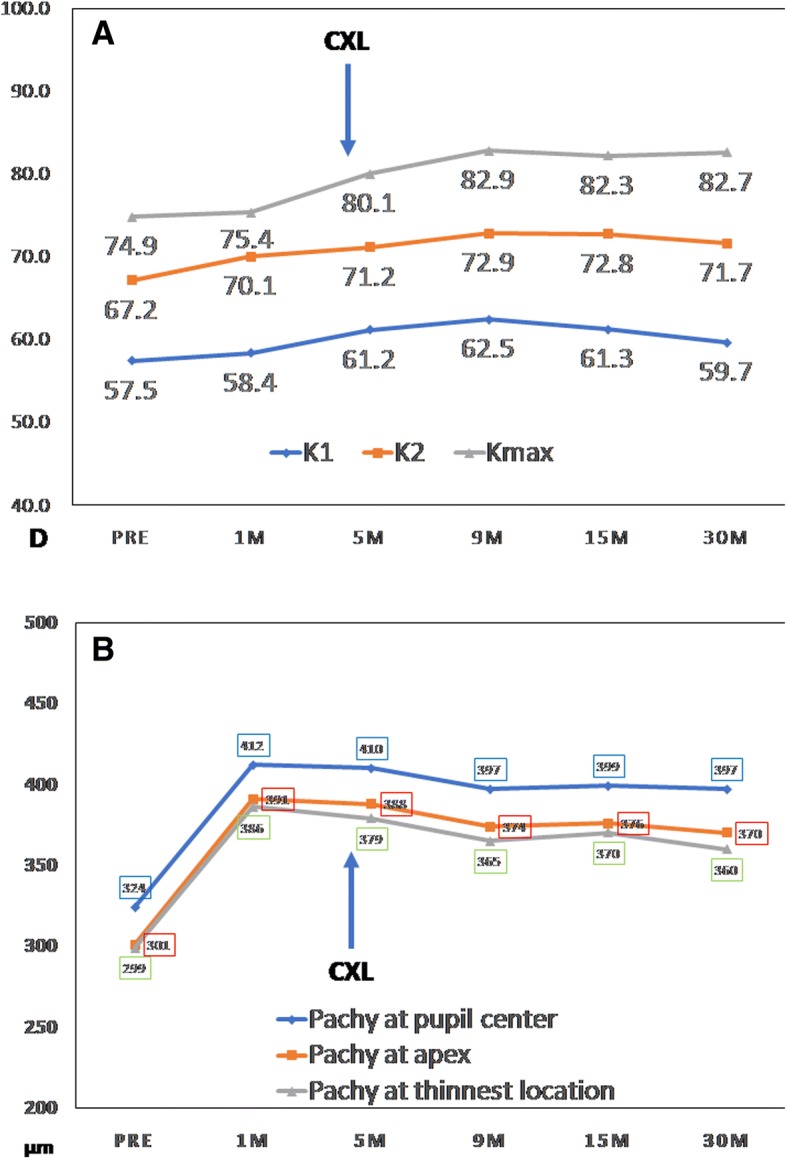
Time-dependent changes of corneal power (**a**) and thickness (**b**), measured with a Pentacam

Differential maps of Scheimpflug corneal topography at 30 months after surgery are shown in Fig. 3. The variation curve of front corneal K1, K2, and Kmax values are shown in Fig. 4a, which increased by 2.1 D, 4.5 D, and 7.8 D, respectively, at postoperative 30 months relative to preoperative values. Mean radius of the posterior curvature was 3.51 mm before surgery and 3.50 mm, 3.47 mm, 3.49 mm, 3.49 mm, and 3.55 at 1, 5, 9, 15, and 30 months post-surgery, respectively. The greatest posterior elevation was + 140 μm before surgery and + 170 μm, + 194 μm, + 154 μm, + 158 μm, and + 141 μm at 1, 5, 9, 15, 30 months post-surgery, respectively, showing an initial increase and then gradual decrease.

Time-dependent changes of corneal thickness measured with a Pentacam (Oculus Optikgeräte, Wetzlar, Germany) preoperatively and 1, 5, 9, 15, 30 months post-op are shown in Fig. 4b. Corneal thickness initially increased, then stabilized at 9 months after the lenticule addition, with a total increase of 73 μm at the pupil center.

**Fig. 5:**
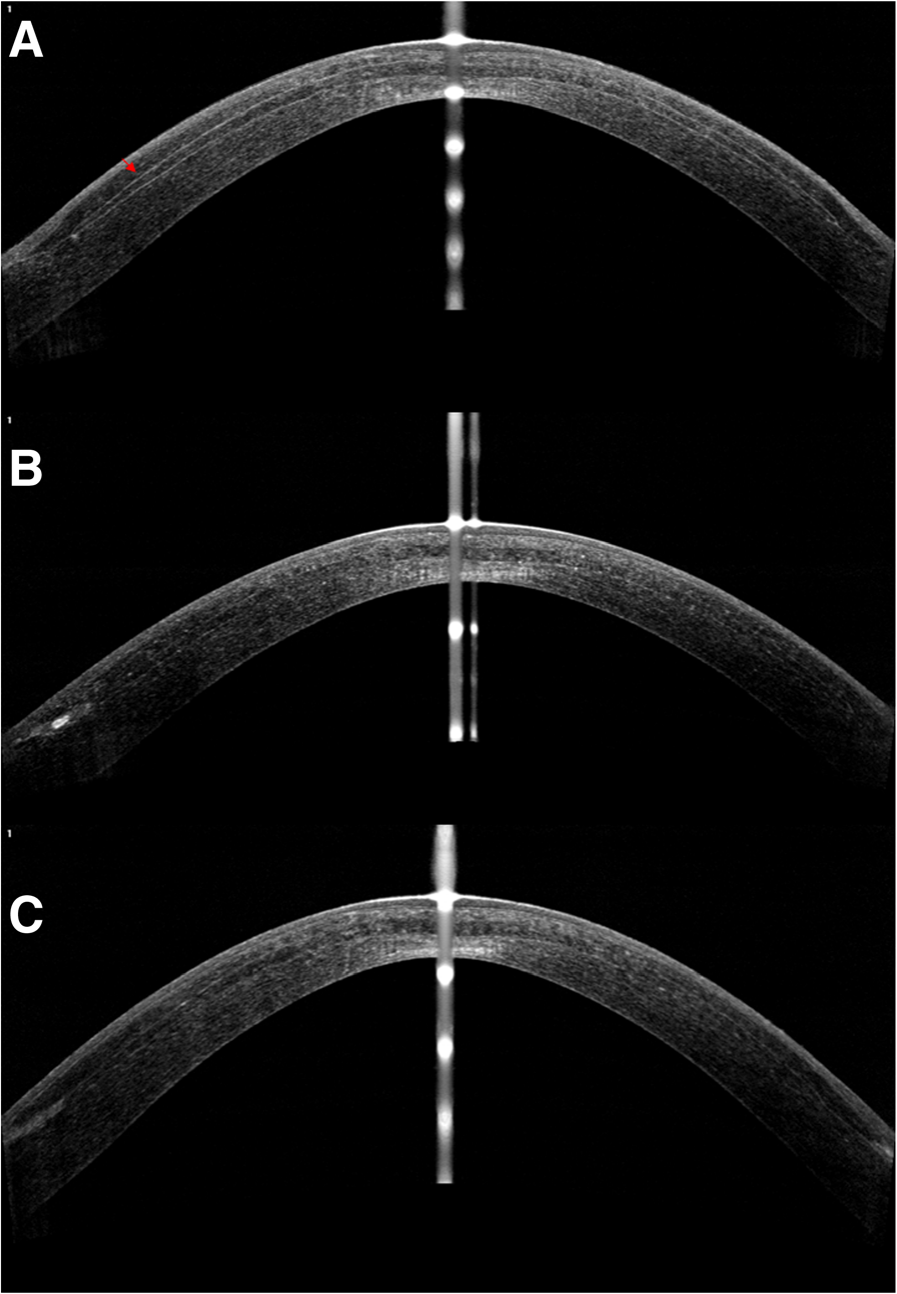
Optical coherence topography showing a clear lenticule at postoperative day 1(**a**). At 5 months follow-up, the lenticule demarcation lines became ambiguous (**b**). At the 30 months postoperatively, the density of the lenticule was similar to that of the surrounding corneal stroma (**c**)

The optical coherence topography (OCT) images (Fig. 5) showed a clear lenticule at postoperative day 1. At 5 months follow-up, the lenticule demarcation lines became ambiguous and the density of the lenticule was similar to that of the surrounding corneal stroma.

**Fig. 6:**
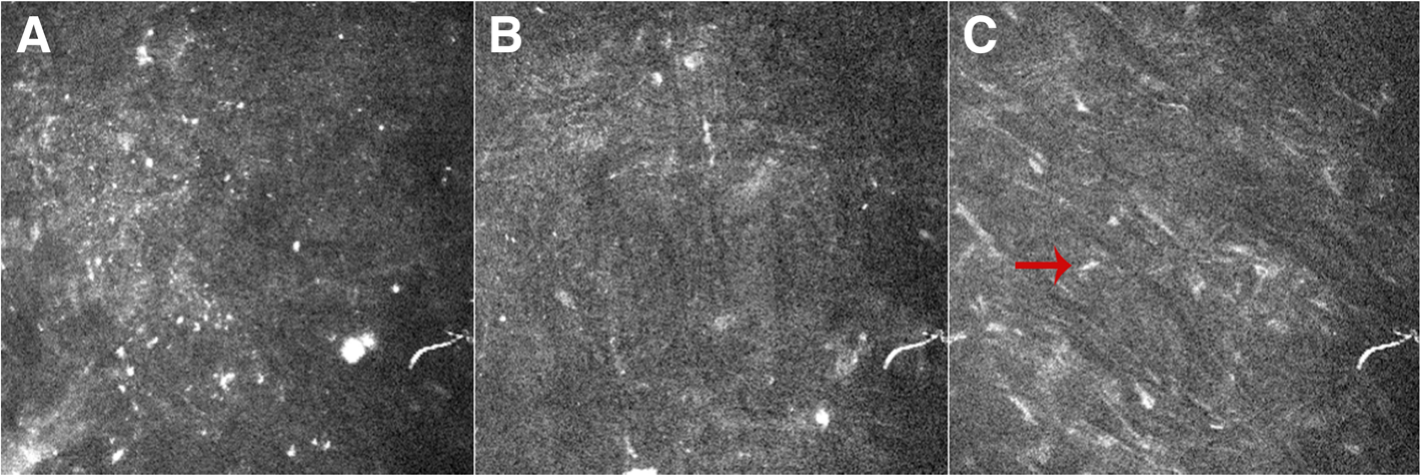
In vivo confocal microscopy presenting the detectable anterior (**a**) and posterior (**b**) lenticule interfaces. Lenticule keratocytes are marked with a red arrow(**c**)

In vivo confocal microscopy (IVCM) showed that both the anterior and posterior lenticule interfaces were detectable, characteristic of an absence or decrease in keratocytes and the presence of small particles of various brightness. Keratocytes in the implanted lenticule demonstrated elongated morphology and a decrease in number (Fig. 6).

## Discussion and conclusions

Post-LASIK corneal ectasia is a rare but serious late postoperative complication. After LASIK, the cornea is structurally weakened, not only by the laser central stromal ablation, but also by the creation of the flap itself. Surgical techniques are evolving for the treatment of both keratoconus and post-LASIK ectasia. Intra-lamellar keratoplasty [1, 10, 11, 13], Bowman layer trasplantation [14, 15] and CXL [16, 17] procedures are developing to decrease corneal ectasia and provide visual rehabilitation with spectacles or contact lenses.

Bilgihan et al. [13], Jiang et al. [11], Mastropasqua et al. [10] and Li et al. [18] reported lamellar keratoplasty for keratoconus and their results showed a significant increment in corneal thickness. In the presented case, we innovatively implanted the lenticule from a myopic SMILE into the corneal stroma of a patient with post-LASIK ectasia. After 30 months, the increase in corneal thickness at the pupil center was 73 μm, which was comparable with the thickness of the implanted lenticule (77 μm). The postoperative posterior elevation (141 μm) was similar with the preoperative one (140 μm). The CDVA increased 2 lines at 1 month postoperatively, which was much faster than in traditional keratoplasty, and this remained stable for the entire follow-up period. In addition, OCT showed the lenticule was well-merged with the adjacent corneal stroma. However, the corneal power and corneal astigmatism increased. It is possible that is due to the refractive error of the lenticule itself, given that the astigmatism of the lenticule (− 2.75 DC) was close to the increase in astigmatism of cornea (2.4 D).

CXL, intended to create a stiffening of the cornea, has been an accepted therapeutic intervention to stabilize corneal ectasia for patients with more than 400 μm of central corneal thickness [6, 10–12]. However, this case was not suitable for CXL due to a corneal thickness under 400 μm. Lenticule addition in this patient demonstrated an increase the corneal thickness, though the initial Kmax (75.4 D) and posterior elevation (170 μm) were high. Combining CXL with lenticule addition is therefore a potential method to prevent the progress of post-LASIK ectasia, and allowed this patient to be a candidate for CXL 4 months after the lenticule addition procedure.

We describe a surgical technique that uses lenticule addition followed by CXL to increase corneal thickness and inhibit the ectatic cornea. The results were promising and without complications. We found lenticule addition to be an efficient technique to increase the corneal thickness, and the subsequent CXL procedure provided corneal stability. With the lenticule addition and CXL technique, the time of procedure was shortened, the complication risk was low, and the donor lenticule was easy to obtain compared to lamellar keratoplasty. These results suggest the efficacy, safety, and stability of lenticule addition with CXL as an emerging surgical technique for post-LASIK ectasia.

Our case is limited to a lenticule extracted from a myopic patient, which may have contributed to the increase in corneal power and astigmatism in this case. Future cases, including hyperopic lenticule addition, will be necessary to confirm the long-term benefits of this surgical technique in the treatment of post-LASIK ectasia with thin corneal thickness.

In conclusion, in post-LASIK ectasia patients with progressive corneal thinning and bulging, performing the lenticule addition prior to CXL may be an effective strategy with reduced risk and shorter treatment time than traditional lamellar keratoplasty.

## Abbreviations

BSS: Balanced salt solution
CDVA: Corrected distance visual acuity
CXL: Corneal cross-linking
IVCM: In vivo confocal microscopy
LASIK: Laser in situ keratomileusis
OCT: Optical coherence topography
RGP: Rigid gas permeable contact lenses
SMILE: Small incision lenticule extraction
UDVA: Uncorrected distance visual acuity
